# COVID-19 and protection measures adopted in rural amazon communities during the first months of the pandemic

**DOI:** 10.17843/rpmesp.2024.413.13017

**Published:** 2024-09-03

**Authors:** Christian Abizaid, Yoshito Takasaki, Oliver T. Coomes

**Affiliations:** 1 Department of Geography & Urban Planning and School of the Environment, University of Toronto, Toronto, Canada. University of Toronto Department of Geography & Urban Planning and School of the Environment University of Toronto Toronto Canada; 2 Graduate School of Economics, University of Tokyo, Tokyo, Japan Graduate School of Economics University of Tokyo Tokio Japón; 3 Department of Geography, McGill University, Montreal, Canada McGill University Department of Geography McGill University Montreal Canada

**Keywords:** COVID-19 Pandemic, Coronavirus infection, Mortality, Rural Populations, Amazon: Peru

## Abstract

**Objectives.:**

To analyze the evolution of COVID-19 in rural populations of Loreto and Ucayali in the early stage of the pandemic.

**Materials and methods.:**

A community-level longitudinal observational study was conducted and based on two rounds of telephone surveys with local authorities of more than 400 indigenous and non-indigenous rural communities in Loreto and Ucayali, in July and August 2020. We collected information on cases and deaths by COVID-19 in their communities, protective measures adopted and if state assistance was received in the early stage of the pandemic. Descriptive statistics allowed us to evaluate the evolution of the pandemic after the initial outbreak and compare the trends of the two regions, as well as between indigenous and non-indigenous populations.

**Results.:**

In July 2020, COVID-19 had reached 91.5% of the communities, although deaths from COVID-19 were reported in 13.0% of the communities, with rural mortality being higher in Ucayali (0.111%) than in Loreto (0.047%) and in non-indigenous communities. By August, prevalence decreased from 44.0% to 32.0% of communities, but became more frequent in indigenous communities, and those in Ucayali. Traveling to the city to receive state bonuses and difficulties maintaining social distancing contributed to the spread.

**Conclusions.:**

Our findings show the evolution of COVID-19 in rural communities and point to important areas of attention in future public policies, for the adoption of protective measures and reconsidering strategies for the distribution of assistance in the face of future pandemics.

## INTRODUCTION

Peru is one of the countries most severely affected by COVID 19 despite having established one of the earliest and longest quarantines in Latin America, and implementing an ambitious social assistance program to help families economically affected by the pandemic ^1^^,^^2^. By the end of June 2020, gradual reopening started, and in September 2020 Peru ranked fifth worldwide in the number of cases ^2^^,^^3^.

COVID-19 arrived to the Peruvian Amazon in mid-March 2020, causing great international concern for the welfare of the rural population, particularly indigenous people ^4^^-^^7^. This concern was later amplified by the coincidence of COVID-19 with dengue ^8^. The situation in Iquitos, the capital of Loreto, was particularly alarming during the first outbreak of the pandemic, with one of the highest infection rates in the world and a collapsed health system ^9^; the situation in Pucallpa was not much better ^4^. However, little is known about how the pandemic evolved in rural jungle communities, which are recognized as among the most vulnerable in the country ^10^.

Studies on the rural situation tend to focus on indigenous communities ^11^^-^^13^. Notably, a study in Amazonas based on data from the Regional Health Directorate (DIRESA) documented 3,919 confirmed cases of COVID-19 among indigenous populations as of July 2020 ^13^^)^ and by June 2021 the number of cases among indigenous people in Loreto and Ucayali based on the population room totaled more than 10,000 ^11^. Less attention has been paid to non-indigenous populations with similar vulnerabilities and, due to their geographic dispersion and access difficulties, also have precarious access to health services ^14^.

Case and mortality data provided by the Ministry of Health (MINSA) ^15^^,^^16^ are extremely valuable, but reflect conditions in more urbanized communities or those with access to health facilities. However, most rural communities lack adequate health facilities or roads, and many are too remote, making it difficult, but vital, to capture the impact of COVID 19 in rural settings. In response to demands from experts and indigenous people, CDC-Peru launched the indigenous population room with COVID-19 based on data from the Epidemiological Surveillance Notification System (NOTI) on confirmed cases and deaths ^17^. While such data have served to monitor the situation among the indigenous population, they are known to be problematic ^11^, and it has not been possible to assess spatial evolution or dynamics at the community level, nor are there comparable data for non-indigenous communities. This study aimed to analyze the evolution of COVID-19 in rural indigenous and non-indigenous communities in Loreto and Ucayali during the early stage of the pandemic in Peru (March to August 2020).

**KEY MESSAGES**

Motivation for the study. To document the evolution of COVID-19 in rural Amazonian populations, which are still little known.

Main findings. COVID-19 spread rapidly through rural communities, initially spreading to mestizo hamlets and later affecting indigenous communities. Rural mortality varied by region and ethnicity. Social distancing was difficult, and travel to receive government vouchers contributed to contagion.

Implications. Identifying the factors that contributed to contagion and the barriers to the adoption of protective measures in rural Amazonian populations will help to face future pandemics.

## MATERIALS AND METHODS

### Study design

This longitudinal observational study was conducted as part of a project that seeks to better understand rural poverty in indigenous and non-indigenous populations in the Peruvian rainforest, known as the Peruvian Amazon Poverty and Rural Livelihoods Project (PARLAP) ^18^^,^^19^. The original PARLAP study consisted of field surveys conducted between September 2012 and March 2014 on community-level characteristics and conditions covering 919 communities along the Amazon, Napo, Pastaza and Ucayali rivers in the regions of Loreto (608 communities) and Ucayali (311 communities) (~92.0% of rural communities in the study area).

### A longitudinal observational study on the evolution of COVID-19 at the community level

Based on the original PARLAP sample, we sought to identify target communities for surveys in rural communities in Loreto and Ucayali. The study focused on rural communities with little or no access to health services in their community based on the original surveys (target communities). Overall, of the 919 communities within the PARLAP study area, 893 were identified as eligible for inclusion, after excluding district capitals and communities with a health center. The surveys were conducted remotely since it was impossible to visit the communities during the health emergency. We sought to contact every possible community within the PARLAP study area, however, due to the suspension of rural public telephone service a few months earlier ^20^ and a deficient radio system, the surveys relied mainly on cell phone contact.

The surveys were designed with simple questions to facilitate answering them over the phone. Given the urgency of capturing the situation and working under difficult and uncertain conditions, a pilot survey was not conducted, but the questions were carefully reviewed to ensure that most of them worked properly.

The baseline telephone survey was conducted in July 2020, between the first and second COVID-19 outbreak, which were defined based on MINSA regional case data (Supplementary Figure 1), covered 469 communities, or 53.0% of the target communities: 369 in Loreto and 100 in Ucayali, being 262 indigenous communities, 206 mestizo hamlets and one settler community (Table 1). The geographic distribution of surveyed communities and COVID-19 cases are shown in Supplementary Figure 2. Subsequently in August 2020 and during the second outbreak, we conducted a follow-up telephone survey covering 435 of the 469 communities in the baseline survey (attrition of 7.0%). In both surveys, information was requested on conditions at the community level, focusing on COVID-19 cases and deaths in the community as a whole, possible causes of infection, protective measures, and received assistance. The baseline survey collected the total number of deaths regardless of cause, and those potentially due to COVID-19 (confirmed and suspected cases) since mid-March of the same year, capturing the first COVID-19 outbreak. The follow-up survey collected data on cases and deaths in the community during the seven days prior to the survey. Together, these surveys capture conditions in rural communities after the first outbreak of the pandemic, as well as possible changes between the two rounds of surveys.

**Table 1:** Characteristics of the communities, Loreto and Ucayali.

	Total	Loreto	Ucayali	Indigenous community	Mestizo hamlet
Number of communities studied	469	369	100	262	206*
Average number of households	78	70	110	64	98
	(153)	(128)	(220)	(87)	(208)
Average number of inhabitants	319	252	568	259	397
	(816)	(322)	(1,638)	(258)	(1,194)
Indigenous (%)	56.0	55.0	58.0	100.0	00.0
Telephone (any) (%)	74.0	69.0	93.0	73.0	75.0
Cell phone (%)	55.0	47.0	86.0	44.0	69.0
Internet (%)	14.0	7.0	40.0	11.0	17.0
Health center (%)	20.0	12.0	47.0	19.0	21.0
Public river transport by boat (%)	68.0	80.0	23.0	66.0	70.0
Public river transport (collective type) (%)	29.0	19.0	65.0	24,0	36.0
Number of communities in follow-up survey	435	344	91	240	194*
Attrition rate (%)	7.2	6.8	9.0	8.4	5.8

Note: Data derived from the baseline sample. Standard deviations are in parentheses. All variables except number of households and number of inhabitants are indicator variables.

* The sample by ethnicity excludes one self-identified settler community. For some variables, the number of observations is smaller than the number of communities due to missing values. The attrition rate corresponds to the percentage of communities in the baseline survey that could not be included in the follow-up survey.

### Basic characteristics of the communities

The characteristics of the communities in the baseline sample are presented in Table 1. Rural communities in the study area self-identify (by their communal authorities) as indigenous (56.0%; regardless of whether they have official recognition as a Native Community) or as mestizo hamlets (44.0%); one community self-identifies as colonists. Mestizo hamlets tend to be located along major rivers, while indigenous communities are located in more remote areas (Supplementary Figure 2A). Rural communities are small (median: 78 households: 319 people) and only 20.0% of them have a health post. The rural population depends mainly on public river transportation.

### Informants

The informants for this study were mostly community authorities (Apu/Community Chief, Lieutenant Governor, Municipal Agent) due to the scarcity of local health personnel and the difficulties in locating them where they existed during the health emergency. Because of their position in the community and considering that communities are typically small, we considered that these individuals were sufficiently informed about the COVID-19 situation in their own community. In May 2020, as quarantine measures were relaxed and the economy began to revive, the population became more mobile and available. Visits were made to ports and markets in Iquitos and Pucallpa to locate potential informants in the target communities and coordinate a telephone survey. Some telephone surveys were also coordinated through a local intermediary in cases where people from the target communities visited a town where an intermediary lived, making it possible to contact local authorities in communities without telephone access.

### Variables

COVID-19 cases and deaths were referred to as those reported in communities by survey informants. Number of deaths in rural communities potentially caused by COVID-19 includes cases suspected but not confirmed by any COVID-19 test (Supplementary Table 1). The analysis is based primarily on the prevalence of COVID-19 in the community in terms of the presence of cumulative cases/deaths between mid-March and July (any/any cases/deaths through July), any cases ongoing at the time of the baseline (July 2020) or follow-up (August 2020) telephone surveys and, any cases (any deaths) in the seven days prior to the follow-up survey.

The degree of adoption (generalized, partial, null) of the following protective measures was considered: hand washing, use of masks, avoiding physical contact, keeping a distance of at least 1 meter, staying at home (unless necessary), avoiding meetings and travel, and restricting access to the outside world in relation to incidence (presence of cases at the time of the baseline survey) and new incidence (presence of cases during the 7 days prior to the follow-up survey). Two types of state aid programs received up to the time of the baseline survey (between March and July) were considered: monetary aid (Bono Familiar Universal) and non-monetary aid (food, medicines, oxygen, masks, disinfectant and soap).

### Data Analysis

The statistical program STATA V15 (StataCorp, TX, USA) was used to analyze data on COVID-19 cases and deaths at the community level derived from baseline and follow-up telephone surveys, capturing possible changes after the first pandemic outbreak. We compared trends in Loreto and Ucayali, as well as between indigenous communities and mestizo hamlets through a descriptive analysis of the proportion of communities with or without COVID-19 cases (deaths) at the time of each survey (baseline and follow-up survey) derived from the respective sample for each survey round (N=469 and N=435). The coincidence of COVID-10 with the adoption of protective measures and receipt of state assistance received was also analyzed descriptively in terms of the proportion of communities with different degrees of adopting the 8 protective measures listed above and of having received different types of assistance, both of which may have affected contagion.

### Ethical considerations

This study was approved by the Human Research Ethics Committee of McGill University (protocol 290-0114). The information was requested from the communities and not particularly to any individual in the community, therefore, only acceptance by the community authorities was requested.

## RESULTS

### Evolution of COVID-19 in rural communities during the early stage of the pandemic: cases and deaths

At the time of the study, COVID-19 had spread throughout rural communities in Loreto and Ucayali. At least one case of COVID-19 (some case, including suspected cases) had been reported in most communities (91.5% in July 2020 in the baseline survey and 94.5% in August 2020 in the follow-up survey), and 12.3% of communities reported some confirmed case by July. In contrast, mortality due to COVID-19 was more limited, 18.0% of communities reported any death due to any cause between mid-March and July 2020, and 13.0% reported any death potentially due to COVID-19.

COVID-19 prevalence decreased, from 44% of communities at the time of the baseline survey to 32.0% by the time of the follow-up survey one month later (Supplementary Figures 2B, 2C). At the same time, 13.0% of the communities in the follow-up sample reported any case of COVID 19 for the first time (Supplementary Figure 2C). One in four communities reported a new case of COVID-19 during the seven days prior to the follow-up survey.

Data on total number of deaths independent of cause and those potentially due to COVID-19 in the baseline survey yield mortality rates of 0.152% and 0.069%, respectively. The latter figure can be considered the upper limit of the mortality rate due to COVID-19 between mid-March and July 2020 (Supplementary Table 1). That is, up to 45.0% of all deaths during this period were potentially caused by COVID-19. The mortality rate varies by ethnicity and by region (Supplementary Table 1), with the rate in Ucayali being more than double the rate in Loreto (0.118% vs. 0.047%) and substantially higher in mestizo hamlets than in indigenous communities (0.118% vs. 0.061%).

**Figure 1:**
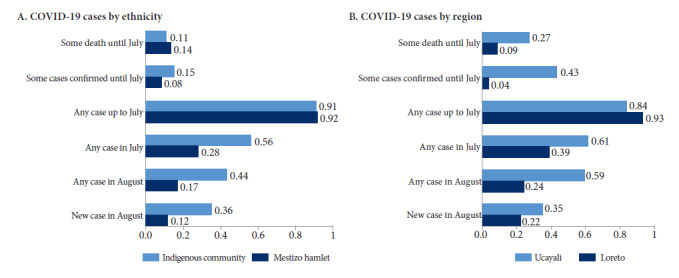
Prevalence and spread of COVID-19 in rural Amazonian communities by ethnicity and region during the first months of the pandemic in Peru.

Note: Any case includes all cases, including confirmed and suspected cases. Up to July refers to any case or death between mid-March and July 2020. In July (or in August) refers to the presence of cases at the time of our baseline survey in July 2020 (or follow-up survey in August). For some variables, the number of observations is less than the number of communities in the sample (reported in Figure 1) due to missing values.

Comparing the proportion of communities with COVID-19 cases and deaths between indigenous communities and mestizo hamlets does not show any substantial difference in the presence of cases and mortality potentially due to COVID-19 in July; with one exception, a larger proportion of indigenous communities reported any confirmed cases until July (Figure 1A). Thereafter, prevalence (any case in July, either suspected or confirmed; any case in August) and incidence (new case in August) increased in indigenous communities, suggesting that in August indigenous communities were more severely affected than mestizo hamlets. Similarly, when comparing the proportion of communities with cases and deaths between communities in Loreto and Ucayali, we found that the presence of any case or death due to COVID 19 was more common in Ucayali than in Loreto (Figure 1B).

### Protective Measures

**Figure 2:**
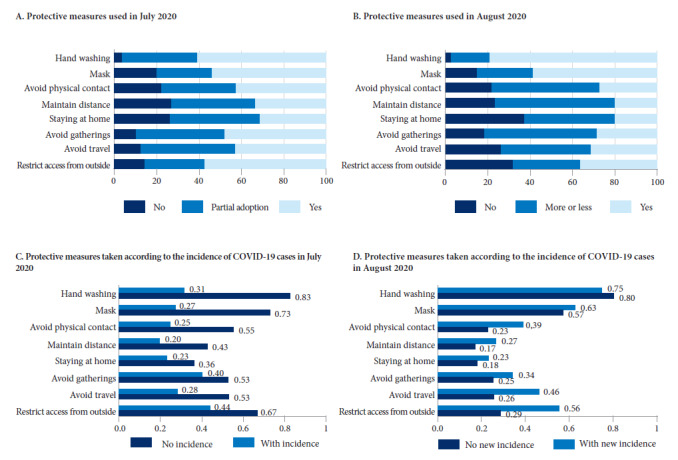
Protective measures in rural Amazonian communities during the first months of the pandemic in Peru.

Notes: In panels A, B, C and D *Keep distance* corresponds to maintaining a sufficient distance of at least 1 meter; *Stay at home* corresponds to not leaving their home unless necessary; Avoid gatherings corresponds to avoiding gatherings with many people; *Avoid travel* corresponds to avoiding people traveling to other places (rural communities or the city); *Restrict access from outside* corresponds to limiting access to the community by people from outside. The other protection measures are obvious. In panels A and B, yes, no and more or less refer to the degree of adoption of each protection measure based on the original survey questions: yes corresponds to widespread adoption, more or less to partial adoption and no to no adoption of the measure. In panels C, *No incidence* and *With incidence* refer to the presence of COVID-19 cases in the community at the time of the baseline survey (July 2020); *No new incidence and With new incidence* refer to the presence of new COVID-19 cases in the community (during the 7 days prior to the follow-up survey). The number of observations is less than the number of communities in the sample (reported in Figure 1) due to missing values.

At the time of the baseline survey, among the protective measures, only hand washing had been widely (yes) or partially (more or less) adopted in the majority of communities (96.0%); mask use and social distancing measures, avoiding physical greetings, social gatherings and travel, keeping a minimum distance, staying at home and restricting entry to the community, were not being adopted in between 10.0% and 27.0% of communities (Figure 2A). Hand washing, use of masks, and establishing community entry restrictions were more widely adopted than other protective measures (~60.0% vs. ~40.0% of communities).

By the time of the follow-up survey, hand washing and mask use became more common relative to the baseline survey in July, but at the same time, adoption of all social distancing measures decreased (Figure 2B).

The baseline survey data indicates that protective measures between mid-March and July were more common in communities without any COVID 19 cases (including suspected cases) in July (Figure 2C). In August, however, social distancing measures were more common in communities reporting any new cases (Figure 2D), possibly to avoid contagion.

### Assistance

By July 2020, people in 97.0% of communities had received some assistance since mid-March. While food (groceries) and monetary (vouchers) assistance was common (over 80% of communities), few communities had received masks, disinfectant, soap or medicine by this time. The state was the most common source of assistance (96.0%) with very little support from other sources (indigenous federations, non-governmental organizations and international organizations).

When assessing data on received assistance and COVID-19, we found that monetary assistance was more frequent in communities with mortality potentially due to COVID-19 between mid-March and July, and in communities with any COVID-19 cases (including suspected cases) in July; there was no such difference in the receipt of non-monetary assistance (mainly food) (Supplementary Figure 3).

## DISCUSSION

The results of this longitudinal observational study on COVID-19 indicate that, despite quarantine measures and access difficulties in the region, COVID-19 spread widely through rural communities in Loreto and Ucayali and mortality varied by region and ethnicity. The adoption of protective measures and social assistance affected contagion.

More than 90% of the communities reported a case of COVID-19. Initially, COVID-19 was more prevalent in the mestizo villages, but later it had a greater effect on the indigenous communities. Indigenous communities were mostly located in more remote areas, indicating that difficulties in access may have been a barrier that delayed but did not eliminate transmission to these areas ^21^. We also found that a significant percentage (45%) of the deaths recorded during the early stage of the pandemic were potentially caused by the virus. However, the mortality rate was lower than in the city (0.270%, based on MINSA data for Loreto and Ucayali; Supplementary Table 1) and the national average (0.186%), with deaths reported in only 15.0% of the communities, most commonly in mestizo hamlets. The low mortality rate reported here is consistent with other studies on indigenous peoples in Ucayali and Amazonas based on official data from MINSA or DIRESA ^13^^,^^22^, which provide some respite from initial concerns, particularly considering the preexisting vulnerabilities of these populations and the enormous difficulties in providing adequate health services in rural areas even during “normal” times ^4^^,^^23^^-^^25^. However, the cultural significance of the death of several indigenous elders should not be discounted ^26^. Studies on the factors that helped prevent infection and mortality in rural communities will be useful in the future. In particular, there is a need to better understand the potential of traditional medicine and local ecological knowledge to complement public policies on pandemics and to improve human health in rural communities ^27^^-^^29^.

This study also points to structural differences between rural communities that merit serious attention. Compared to Loreto, communities in Ucayali had a much higher COVID-19 mortality rate (0.118% vs. 0.047%) and a higher prevalence in both cases and mortality. Initially, the mortality rate due to COVID-19 was higher in mestizo villages than in indigenous communities, although COVID-19 became more frequent in indigenous communities during the second outbreak. Further studies are needed to better understand the factors underlying such differences, although it is clear that all rural communities (both indigenous and mestizo) require more serious attention and greater involvement to improve their health ^14^^,^^27^^-^^29^.

In terms of protective measures, we also found a higher rate of hand washing and use of masks in relation to other social distancing measures (60.0% vs. 40.0%), which indicates that social distancing is difficult in rural communities, possibly due to social and cultural norms; which has also been reported in other studies ^12^^,^^21^. There is a need to better understand the potential and barriers to the adoption of socially costly protective measures to reduce contagion, such as in this case, social distancing and avoidance of meetings. Further studies are needed to identify factors affecting the adoption of protective measures, which may guide interventions that promote their adoption and respect underlying sociocultural norms ^12^^,^^27^^,^^30^.

Although state assistance programs reached most communities and helped the population, the receipt of state vouchers (82.0% received vouchers) was associated with potential COVID-19 deaths (11.0% more of the communities), the same relationship was not found with the provision of non-monetary assistance (mainly food). This is attributed, not to the type of assistance per se, but to the mode of delivery. While food was delivered directly to the communities, people had to travel to Iquitos, Pucallpa, or district capitals to collect their vouchers. As other studies in Loreto and Ucayali have suggested, these trips contributed unanticipatedly to contagion in rural communities ^4^^,^^12^^,^^13^^,^^21^. Alternative public policies, both new policies and improvements to current policies, are required, namely: alternative protocols for providing social assistance, such as mobile money and the multifunctional itinerant platforms of the Ministry of Social Inclusion, and the provision of information and communication technology resources in rural areas. More applied research is needed to identify promising and feasible approaches in the local context.

Our study has four main limitations. First, the sample of communities is not representative of the study area because of difficulties in contacting all target communities by cell phone; external validity problems have been common with telephone surveys during the pandemic. Then, the possibility of measurement error cannot be ruled out, although community-level telephone surveys reduce the possibility of reporting bias (e.g., social desirability bias) relative to household telephone surveys. Besides, because of poor access to health facilities and COVID-19 testing, the number of confirmed COVID-19 cases reported in surveys is incomplete. The number of reported deaths should be more reliable, although participants’ perceptions of whether deaths were caused by COVID-19 may be inaccurate; thus, the study focuses on the presence of cases and deaths in communities as crude measures of the situation. The collected data does not allow us to study the dynamics at the household or individual levels. Finally, COVID-19 evolution could not be captured in some communities due to the loss of communities in the follow-up survey.

In conclusion, COVID-19 spread widely through rural communities in the jungle, although the mortality rate was lower than in the city, and its impact was greater in indigenous communities and in those of Ucayali. Sociocultural barriers to the adoption of social distancing measures and the need to travel to collect state bonds contributed to contagion in these populations. In addition to the need for an intercultural and more inclusive health approach to strengthen the rural health system ^22^^,^^24^^,^^29^^,^^30^, it is necessary to better understand the factors that affect the adoption of protective measures, as well as to rethink the modes of distribution of state aid programs to prevent infection in future pandemics. Overall, priority should be given to establishing reliable data collection systems that reflect conditions in rural communities in order to inform effective policies.
