# Supplementary material for: COVID-19 and protection measures adopted in rural amazon communities during the first months of the pandemic
Source: Rev Peru Med Exp Salud Publica. 2024 Sep 3;41(3):239–46. doi: 10.17843/rpmesp.2024.413.13017 (PMC11495933; doi:10.17843/rpmesp.2024.413.13017)
Supplement: Supplementary material. — Available in the electronic version of the RPMESP. [file rpmesp-41-03-13017-s001.docx]

| **Materiales Suplementarios**  **Tabla Suplementaria 1.** Mortalidad en Loreto y Ucayali. | | | | | | | |
| --- | --- | --- | --- | --- | --- | --- | --- |
|  |  |  |  |  |  |  |  |
|  |  |  | Total | Loreto | Ucayali | Comunidad indígena | Caserío mestizo |
| Número de comunidades estudiadas | |  | 469 | 369 | 100 | 262 | 206 |
|  |  |  |  |  |  |  |  |
| **Mortalidad en comunidades rurales** | |  |  |  |  |  |  |
| Número de muertes en comunidades rurales potencialmente causadas por COVID-19 - encuestas telefónicas COVID-PARLAP | |  | 92 | 44 | 48 | 43 | 49 |
|  | Tasa de mortalidad rural (%) |  | 0,069 | 0,047 | 0,118 | 0,061 | 0,118 |
|  | Población en comunidades rurales (miles de habitantes) |  | 134 | 93 | 41 | 68 | 66 |
|  |  |  |  |  |  |  |  |
| **Mortalidad a nivel regional** | |  |  |  |  |  |  |
| Número de muertes por COVID-19 - MINSA | |  | 3 730 | 2 486 | 1 244 | - | - |
|  | Tasa de mortalidad (%) |  | 0,270 | 0,281 | 0,251 | - | - |
|  | Población total (miles de habitantes) |  | 1 380 | 884 | 496 | - | - |
| Notas: La muestra por etnia excluye una comunidad autoidentificada como colono. Para algunas variables, el número de observaciones es menor que el número de comunidades debido a valores faltantes. El número de muertes y la tasa de mortalidad en porcentaje al momento de la encuesta de línea base (julio 2020). Muertes en comunidades rurales potencialmente causadas por COVID-19 incluye aquellas debidas a casos sospechosos no confirmados por alguna prueba COVID. Población en comunidades rurales representa el número total de habitantes reportado en las comunidades rurales incluidas en el estudio al momento de la encuesta de línea base. El número de muertes en los datos del Ministerio de Salud (MINSA) son muertes desde mediados de marzo de 2020 hasta el último día de la encuesta de base (2 de agosto de 2020) (MINSA 2021a). Población total representa el número total de habitantes en los departamentos de Loreto y Ucayali basada en el Censo de Población y Vivienda de 2017 levantado por el Instituto Nacional de Estadística e Informática (INEI, 2018). | | | | | | | |


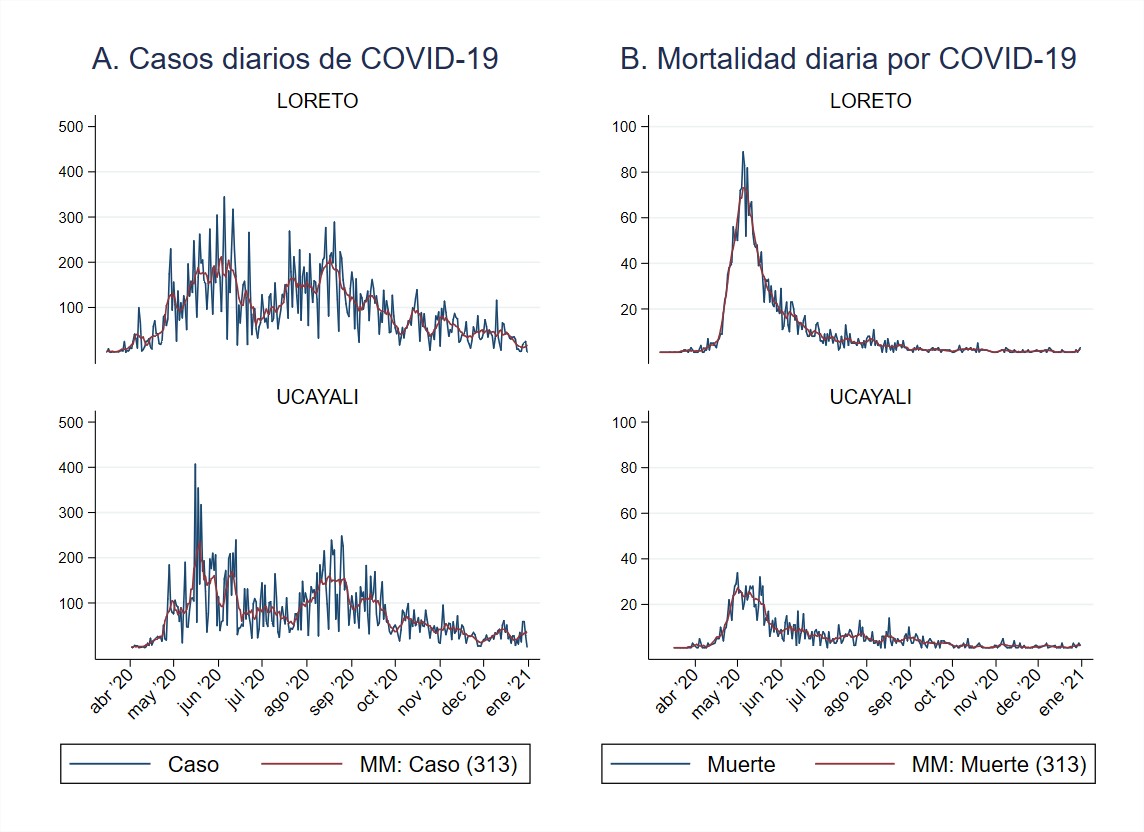


Notas: Construida en base a datos del Ministerio de Salud (MINSA) disponibles a través de la Plataforma de Datos Abiertos del Estado Peruano actualizados a enero de 2021. Casos de COVID-19 (panel A) basados en el *Dataset* de *casos positivos por COVID-19* (MINSA 2021a)*,* mortalidad (panel B) basada en *Dataset de fallecidos por COVID-19* (MINSA 2021b)*.* Caso y muerte se refieren al número de casos y muertes diarios. *MM: caso* y *MM: muerte* corresponden a la media móvil central de casos y muertes en un periodo de siete días (incluyendo tres días anteriores y tres días posteriores a cada fecha). En el panel A se pueden observar los dos brotes de COVID-19 mencionados en el estudio. El brote inicial, entre marzo y junio de 2020, y el segundo brote comenzado en agosto de 2020.

**Figura Suplementaria 1.** Tendencias regionales de COVID-19 en Loreto y Ucayali.


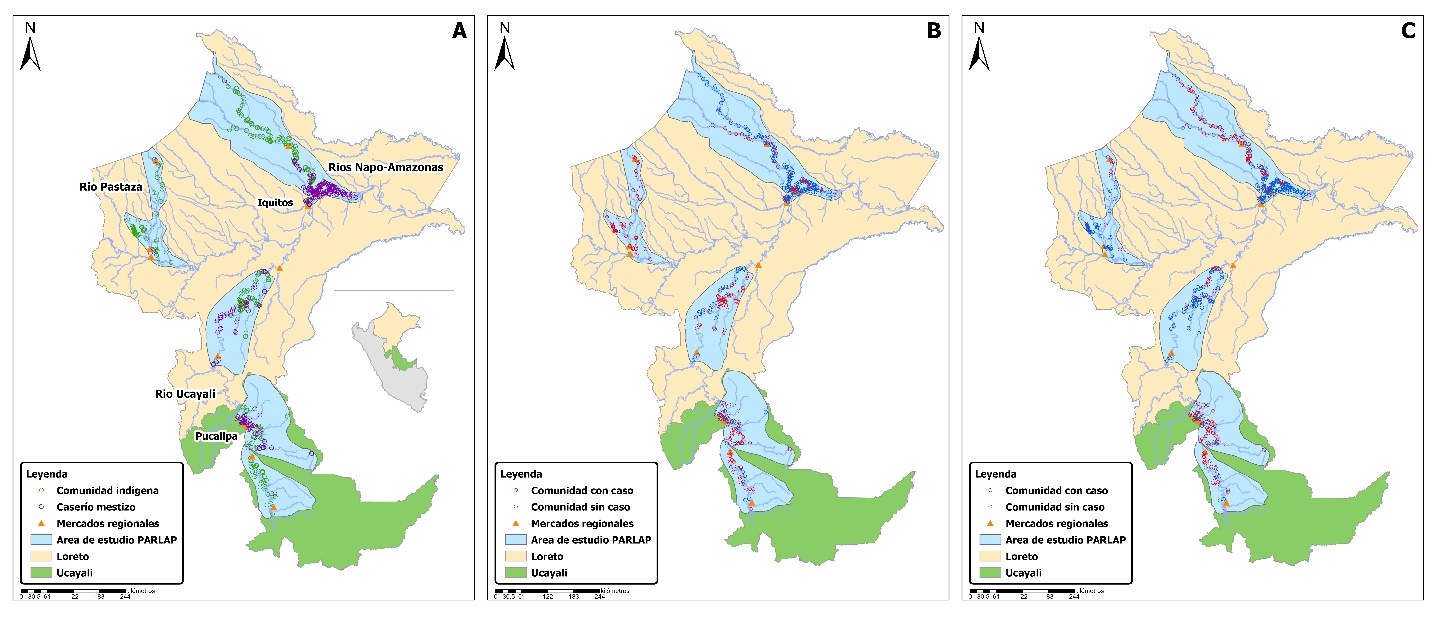


Panel A: Comunidades rurales incluidas en el estudio, basado en la encuesta de base (julio 2020). Se distinguen comunidades indígenas y no-indígenas (caseríos mestizos); una comunidad que se autoidentifica como colonos fue excluida. Panel B: Prevalencia de casos de COVID-19 en las comunidades rurales en julio de 2020. Panel C: Prevalencia de casos de COVID-19 en las comunidades rurales en agosto de 2020.

**Figura Suplementaria 2.** Comunidades encuestadas y prevalencia de COVID-19.


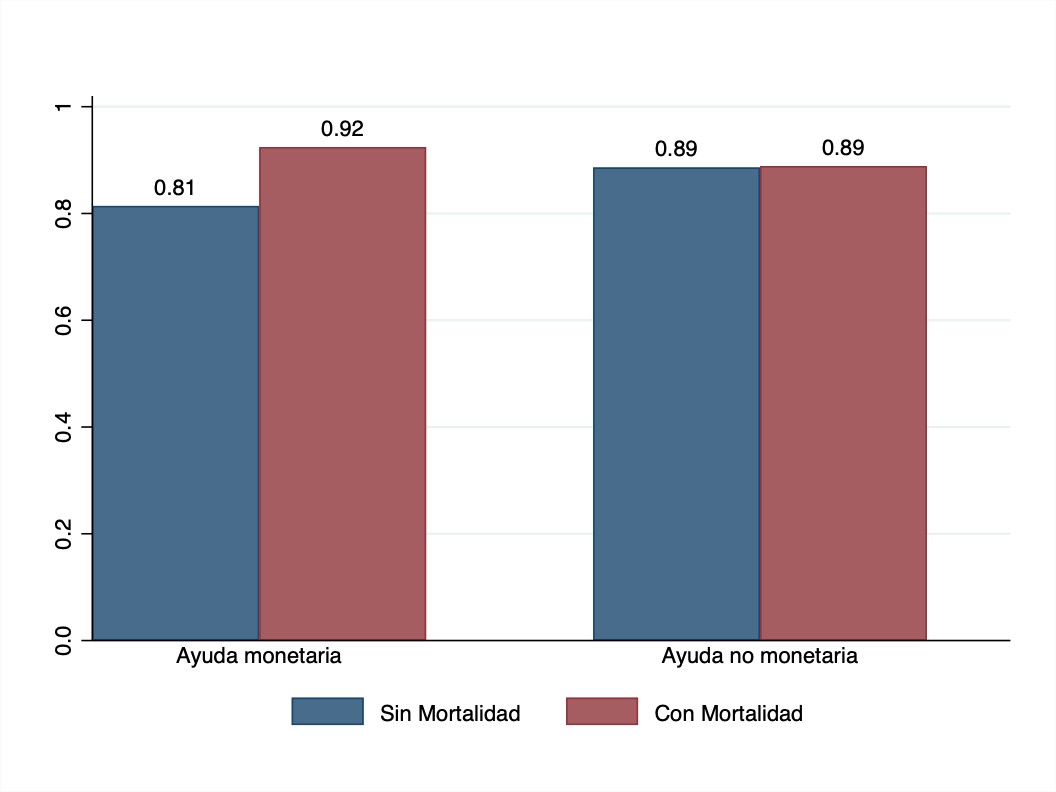


Notas: Asistencia monetaria incluye programas de ayuda social para apoyar a las familias ante las dificultades de la emergencia económica (por ej. Bono Familiar Universal). Beneficiarios tuvieron que viajar a la ciudad para cobrar sus bonos. Asistencia no monetaria incluye víveres, medicinas, oxigeno, mascarillas, desinfectante y jabón. Este tipo de asistencia fue predominantemente llevada a las comunidades para su distribución y uso. El tamaño de las muestras es N=419 y N=432, para asistencia monetaria y asistencia no monetaria respectivamente.

**Figura Suplementaria 3.** Mortalidad en comunidades rurales por tipo de asistencia estatal entre marzo y julio de 2020.

**Referencias bibliográficas**

INEI. Censos Nacionales 2017: XII de Población, VII de Vivienda. Resultados definitivos. Lima: Instituto Nacional de Estadística e Informática; 2018.

Ministerio de Salud (MINSA). [Internet]. Dataset de fallecidos por COVID-19. Plataforma de Datos Abiertos. Centro Nacional de Epidemiología, Prevención y Control de Enfermedades – MINSA, Gobierno del Perú. 2021a. [citado el 14 de junio de 2023]. Disponible en: <https://www.datosabiertos.gob.pe/dataset/fallecidos-por-covid-19-ministerio-de-salud-minsa>

Ministerio de Salud (MINSA). [Internet]. Casos positivos por COVID-19. MINSA. Plataforma de Datos Abiertos. Gobierno del Perú. 2021b. Disponible en: [https://www.datosabiertos.gob.pe/dataset/casos-positivos-por-covid-19-ministerio-de-salud-minsa](https://www.datosabiertos.gob.pe/dataset/casos-positivos-por-covid-19-ministerio-de-salud-minsa%20%0d)
