# Supplementary material for: COVID-19 and protection measures adopted in rural amazon communities during the first months of the pandemic
Source: Rev Peru Med Exp Salud Publica. 2024 Sep 3;41(3):239–46. doi: 10.17843/rpmesp.2024.413.13017 (PMC11495933; doi:10.17843/rpmesp.2024.413.13017)
Supplement: Translation

# Translation: COVID-19 y medidas de protección adoptadas en comunidades rurales amazónicas durante los primeros meses de la pandemia

**Authors:** Christian Abizaid, Yoshito Takasaki, Oliver T. Coomes

**Affiliations:** 1 Departamento de Geografía & Urbanismo y Escuela de Medio Ambiente, University of Toronto, Toronto, Canadá; 2 Escuela de Posgrado en Economía, University of Tokyo, Tokio, Japón.; 3 Departamento de Geografía, McGill University, Montreal, Canadá.

**Keywords:** Pandemia de COVID-19, Infecciones por Coronavirus, Mortalidad, Población Rural, Amazonía, Perú

## Abstract

**Objetivos.:**

Analizar la evolución del COVID-19 en poblaciones rurales de Loreto y Ucayali en la etapa temprana de la pandemia.

**Materiales y métodos.:**

Se realizó un estudio observacional longitudinal a nivel de comunidades basado en dos rondas de encuestas telefónicas con autoridades locales de más de 400 comunidades rurales indígenas y no-indígenas en Loreto y Ucayali, en julio y agosto de 2020, para recopilar información sobre casos y muertes por COVID-19 en sus comunidades, medidas de protección adoptadas y la recepción de asistencia estatal en la etapa temprana de la pandemia. Estadísticas descriptivas permiten evaluar la evolución de la pandemia después del brote inicial y comparar las tendencias de las dos regiones, así como entre poblaciones indígenas y no-indígenas.

**Resultados.:**

En julio de 2020, el COVID-19 había llegado al 91,5% de las comunidades, aunque se reportaron muertes por COVID-19 en 13,0% de las comunidades, siendo la mortalidad rural mayor en Ucayali (0,111%) que en Loreto (0,047%) y en comunidades no-indígenas. Para agosto, la prevalencia disminuyó de 44,0% a 32,0% de comunidades, pero se volvió más frecuente en las comunidades indígenas, y aquellas en Ucayali. Viajar a la ciudad para recibir bonos estatales y las dificultades para mantener el distanciamiento social contribuyeron al contagio.

**Conclusiones.:**

Los hallazgos mostraron la evolución del COVID-19 en comunidades rurales y señalan áreas importantes de atención en futuras políticas públicas, para la adopción de medidas de protección y reconsiderar estrategias para la distribución de asistencia ante pandemias futuras.

## INTRODUCCIÓN

El Perú figura entre los países más severamente afectados por el COVID-19 a pesar de haber establecido una de las primeras y más largas cuarentenas en América Latina, y de implementar un ambicioso programa de asistencia social para ayudar a familias afectadas económicamente por la pandemia ^1^^,^^2^. Para fines de junio de 2020 se inició una reapertura gradual y en setiembre del mismo año, el Perú se situó en el quinto lugar a nivel mundial en términos de casos ^2^^,^^3^.

El COVID-19 llegó a la Amazonía Peruana a mediados de marzo de 2020, generando gran preocupación internacional por el bienestar de la población rural, especialmente de los pueblos indígenas ^4^^-^^7^. A tal preocupación se sumó posteriormente la coincidencia de COVID-19 con el dengue ^8^. La situación en Iquitos, la capital de Loreto, fue especialmente alarmante durante el primer brote de la pandemia, reportándose una de las tasas de contagio más altas del mundo y un sistema de salud colapsado ^9^; la situación en Pucallpa no fue mucho mejor ^4^. Sin embargo, se conoce poco sobre como evolucionó la pandemia en las comunidades rurales de la selva, las cuales se reconocen dentro de las más vulnerables del país ^10^.

Los estudios sobre la situación rural tienden a enfocarse a las comunidades indígenas ^11^^-^^13^. A notar, un estudio en Amazonas basado en datos de la Dirección Regional de Salud (DIRESA) documentó 3 919 casos confirmados de COVID-19 entre poblaciones indígenas a julio de 2020 ^13^ y para junio de 2021 el número de casos entre indígenas en Loreto y Ucayali en base a la sala poblacional sumó más de 10 000 ^11^. Se ha prestado menor atención a poblaciones no-indígenas que presentan vulnerabilidades similares y que, por su dispersión geográfica y dificultades de acceso, tienen también un acceso precario a servicios de salud ^14^.

Los datos sobre casos y mortalidad proporcionados por el Ministerio de Salud (MINSA) ^15^^,^^16^ son sumamente valiosos, pero reflejan condiciones en comunidades más urbanizadas o aquellas con establecimientos de salud. Sin embargo, la mayoría de las comunidades rurales carecen de establecimientos de salud o vías de comunicación adecuadas, y muchas son demasiado remotas, por lo que es difícil pero vital capturar los impactos del COVID-19 en el ámbito rural. En respuesta a demandas de expertos y de los pueblos originarios, CDC-Perú lanzó la sala de población indígena con COVID-19 basada en datos del Sistema de Notificación de la Vigilancia Epidemiológica (NOTI) sobre casos confirmados y muertes ^17^. Si bien, dichos datos han servido para monitorear la situación entre la población indígena, se sabe que son problemáticos ^11^, y no ha sido posible investigar la evolución espacial o las dinámicas a nivel de comunidades, ni tampoco existen datos comparables para comunidades no-indígenas. El objetivo principal de este estudio fue analizar la evolución del COVID-19 en comunidades rurales indígenas y no-indígenas de Loreto y Ucayali durante la etapa temprana de la pandemia en Perú (marzo a agosto de 2020).

**MENSAJES CLAVE**

Motivación para realizar el estudio. Documentar la evolución del COVID-19 en poblaciones rurales Amazónicas, las cuales son aún poco conocidas.

Principales hallazgos. El COVID-19 se propagó rápidamente por las comunidades rurales, extendiéndose inicialmente por los caseríos mestizos y después afectando a comunidades indígenas. La mortalidad rural varió por región y por etnia. El distanciamiento social fue difícil y los viajes para recibir bonos estatales contribuyeron al contagio.

Implicancias. Conocer los factores que contribuyeron al contagio y las barreras para la adopción de medidas de protección en poblaciones rurales Amazónicas ayudarán a enfrentar pandemias futuras.

## MATERIALES Y MÉTODOS

### Diseño del estudio

El presente estudio observacional longitudinal fue realizado como parte de un proyecto que busca entender mejor la pobreza rural en poblaciones indígenas y no-indígenas de la selva peruana, conocido como el Proyecto sobre Pobreza y Formas de Vida Rural en la Amazonia Peruana, (PARLAP, por sus siglas en inglés) ^(^^18^^,^^19^. El estudio original PARLAP, consistió en encuestas de campo realizadas entre setiembre de 2012 y marzo de 2014, sobre características y condiciones a nivel comunitario abarcando 919 comunidades a lo largo de los ríos Amazonas, Napo, Pastaza y Ucayali en las regiones de Loreto (608 comunidades) y Ucayali (311 comunidades) (~92,0% de las comunidades rurales en el área de estudio).

### Un estudio observacional longitudinal sobre la evolución de COVID-19 a nivel de comunidad

En base a la muestra original PARLAP se buscó identificar a comunidades meta para levantar encuestas en comunidades rurales de Loreto y Ucayali. El estudio se enfocó en comunidades rurales con acceso precario o nulo a servicios de salud en su comunidad en base a las encuestas originales (comunidades meta). En total, de las 919 comunidades dentro del área de estudio PARLAP, se identificaron 893 reuniendo las condiciones de inclusión, después de excluir a capitales de distrito y comunidades con un centro de salud. Siendo imposible visitar las comunidades durante la emergencia sanitaria fue necesario realizar las encuestas de manera remota. Se contempló contactar a toda comunidad posible dentro del área de estudio PARLAP, sin embargo, debido a la suspensión del servicio telefónico público rural unos meses antes ^20^ y a un sistema de radiofonía deficiente, las encuestas dependieron principalmente del contacto por teléfono móvil.

Se diseñaron las encuestas con preguntas simples para facilitar su respuesta por teléfono. Ante la urgencia de capturar la situación y trabajando en condiciones difíciles e inciertas no se llevó a cabo una encuesta piloto, pero se hizo una verificación cuidadosa para asegurar que la mayoría de las preguntas funcionara.

La encuesta telefónica de base fue realizada en julio de 2020, entre el primer y el segundo brote de COVID-19, definidos en base a los datos regionales sobre casos del MINSA (figura suplementaria 1), abarcó 469 comunidades, o 53,0% de las comunidades meta (369 en Loreto y 100 en Ucayali, siendo 262 comunidades indígenas, 206 caseríos mestizos y una comunidad de colonos (Tabla 1), la distribución geográfica de las comunidades encuestadas y de casos de COVID-19 se muestran en la figura suplementaria 2. Posteriormente en agosto de 2020 y durante el segundo brote, se realizó una encuesta telefónica de seguimiento que abarcó 435 de las 469 comunidades de la encuesta de base (atrición de 7,0%). En ambas encuestas, se solicitó información sob re las condiciones a nivel de comunidad, enfocándose en casos y muertes por COVID-19 en la comunidad en su conjunto, posibles causas de contagio, medidas de protección y asistencia recibida. La encuesta de base recopiló el número total de muertes independientemente de la causa, y de aquellas que potencialmente se debieron al COVID-19 (casos confirmados y presuntos) desde mediados de marzo del mismo año, capturando el primer brote del COVID-19. La encuesta de seguimiento recopiló datos sobre casos y muertes en la comunidad durante los siete días previos a la encuesta. En conjunto, estas encuestas capturan las condiciones en las comunidades rurales después del primer brote de la pandemia, así como posibles cambios entre las dos rondas de encuestas.

**Tabla 1:** Características de las comunidades, Loreto y Ucayali.

	Total	Loreto	Ucayali	Comunidad indígena	Caserío mestizo
Número de comunidades estudiadas	469	369	100	262	206*
Número de hogares en promedio	78	70	110	64	98
	(153)	(128)	(220)	(87)	(208)
Número de habitantes en promedio	319	252	568	259	397
	(816)	(322)	(1 638)	(258)	(1 194)
Indígena (%)	56,0	55,0	58,0	100,0	00,0
Teléfono (cualquiera) (%)	74,0	69,0	93,0	73,0	75,0
Teléfono móvil (%)	55,0	47,0	86,0	44,0	69,0
Internet (%)	14,0	7,0	40,0	11,0	17,0
Puesto de salud (%)	20,0	12,0	47,0	19,0	21,0
Transporte público fluvial tipo lancha (%)	68,0	80,0	23,0	66,0	70,0
Transporte público fluvial tipo colectivo (%)	29,0	19,0	65,0	24,0	36,0
Número de comunidades en encuesta de seguimiento	435	344	91	240	194*
Tasa de atrición (%)	7,2	6,8	9,0	8,4	5,8

Nota: Datos derivados de la muestra de línea base. Las desviaciones estándar están entre paréntesis. Todas las variables, excepto número de hogares y número de habitantes, son variables indicadoras.

* La muestra por etnia excluye una comunidad autoidentificada como colono. Para algunas variables, el número de observaciones es menor que el número de comunidades debido a valores faltantes. La tasa de atrición corresponde al porcentaje de comunidades en la encuesta de base que no pudieron ser incluidas en la encuesta de seguimiento.

### Características básicas de las comunidades

Las características de las comunidades en la muestra de base se presentan en la tabla 1. Las comunidades rurales en el área de estudio se autoidentifican (por sus autoridades comunales) como indígenas (56,0%; sin importar si tienen reconocimiento oficial como *Comunidad Nativa*) o como caseríos mestizos (44,0%); una comunidad se autoidentifica como colonos). Los caseríos mestizos tienden a estar situados a lo largo de los ríos principales, mientras que las comunidades indígenas se encuentran en áreas más remotas (figura suplementaria 2A). Las comunidades rurales son pequeñas (medias: 78 hogares: 319 personas) y solamente el 20,0% de ellas cuenta con un puesto de salud. La población rural depende principalmente del transporte público fluvial.

### Informantes

Los informantes para este estudio fueron en su mayoría autoridades comunitarias (Apu/Jefe de la Comunidad, Teniente Gobernador, Agente Municipal) debido a la escasez del personal de salud local y a las dificultades para ubicarlo donde si existía durante la emergencia sanitaria. Por su cargo en la comunidad y considerando que las comunidades son típicamente pequeñas, se juzgó que estas personas estaban suficientemente informadas sobre de la situación sobre COVID-19 en su propia comunidad. En mayo de 2020, conforme se fueron relajando las medidas de cuarentena y comenzó a reactivarse la economía, la población contó con mayor movilidad y estuvo más disponible. Se visitaron los puertos y mercados en Iquitos y Pucallpa para ubicar a informantes potenciales de las comunidades meta y coordinar una encuesta telefónica. También se coordinaron algunas encuestas telefónicas a través de un intermediario local en casos donde personas de las comunidades meta visitaban un pueblo donde vivía algún intermediario, permitiendo contactar a autoridades locales de comunidades sin acceso telefónico.

### Variables

Se denominó como casos y muertes por COVID-19 a aquellos reportados en las comunidades por los informantes de las encuestas. Número de muertes en comunidades rurales potencialmente causadas por COVID-19 incluye casos sospechosos, pero no confirmados por alguna prueba de COVID-19 (tabla suplementaria 1). El análisis se basa principalmente en la prevalencia de COVID-19 en la comunidad en términos de la presencia de casos/muertes acumuladas entre mediados de marzo y julio (algún/alguna caso/muerte hasta julio); algún caso vigente al momento de las encuestas telefónicas de base (julio de 2020) o de seguimiento (agosto de 2020) y; algún caso (alguna muerte) en los siete días previos a la encuesta de seguimiento.

Se consideró el grado de adopción (generalizada, parcial, nula) de las siguientes medidas de protección: lavado de manos, uso de mascarilla, evitar contacto físico, mantener distancia de al menos 1 metro, quedarse en casa (a menos que sea necesario), evitar reuniones y viajes, y la restricción al acceso de fuera en relación con incidencia (presencia de casos al momento de la encuesta de base) y nueva incidencia (presencia de casos durante los 7 días previos a la encueta de seguimiento). Se consideran dos tipos de programas de ayuda estatal recibidos hasta el momento de la encuesta de base (entre marzo-julio): ayuda monetaria (Bono Familiar Universal) y ayuda no monetaria (víveres, medicinas, oxígeno, mascarillas, desinfectante y jabón).

### Análisis de Datos

Se utilizó el programa estadístico STATA V15 (StataCorp, TX, E.E. U.U.) para analizar los datos sobre casos y muertes por COVID-19 a nivel de comunidad derivados de las encuestas telefónicas de base y de seguimiento, capturando posibles cambios después del primer brote de la pandemia. Se comparan tendencias en Loreto y Ucayali, así como entre comunidades indígenas y caseríos mestizos por medio de un análisis descriptivo de la proporción de comunidades con o sin casos (muertes) por COVID-19 al momento de cada encuesta (encuesta de base y encuesta de seguimiento) derivadas de la muestra respectiva para cada ronda de encuestas (N=469 y N=435). Se analiza descriptivamente también la coincidencia de COVID-10 con la adopción de medidas de protección y la recepción de asistencia estatal recibida en términos de la proporción de comunidades con diferentes grados de la adopción de las 8 medidas de protección listadas arriba y de haber recibido distintos tipos de asistencia, los cuales pudieron afectar el contagio.

### Consideraciones éticas

Este estudio fue aprobado por el Comité de Ética de Investigación con Seres Humanos de la Universidad de McGill (protocolo 290-0114). Debido a que la información solicitada fue a nivel de comunidad y no particular a ningún individuo de la comunidad, solo se solicitó la aceptación por parte de las autoridades comunitarias. 

## RESULTADOS

### Evolución del COVID-19 en comunidades rurales durante la etapa temprana de la pandemia: casos y muertes

Al momento del estudio, el COVID-19 se había extendido por las comunidades rurales de Loreto y Ucayali: al menos un caso de COVID-19 (algún caso, incluidos casos presuntos) había sido reportado en la mayoría de las comunidades (91,5% en julio de 2020 en la encuesta de base y 94,5% en agosto de 2020 en la encuesta de seguimiento), y el 12,3% de las comunidades reportaron algún caso confirmado hasta julio. En cambio, la mortalidad debida al COVID-19 fue más limitada: el 18,0% de las comunidades registró alguna muerte debida a cualquier causa entre mediados de marzo y julio de 2020, y el 13,0% reportó alguna muerte potencialmente debida al COVID-19.

La prevalencia del COVID-19 disminuyó, de estar presente en un 44% de las comunidades al momento de la encuesta de base a 32,0% para el momento de la encuesta de seguimiento un mes más tarde (figuras suplementarias 2B, 2C). Al mismo tiempo, de las comunidades en la muestra de seguimiento, el 13,0% reportó por primera vez algún caso de COVID-19 (figura suplementaria 2C). Una de cada cuatro comunidades reportó un nuevo caso de COVID-19 durante los siete días anteriores a la encuesta de seguimiento.

Datos sobre número total de muertes independiente de la causa y aquellas potencialmente debidas al COVID-19 en la encuesta de base arrojan tasas de mortalidad de 0,152% y 0,069%, respectivamente. Esta última cifra puede considerarse el límite superior de la tasa de mortalidad debida al COVID-19 entre mediados de marzo y julio de 2020 (tabla suplementaria 1). Es decir, hasta el 45,0% de todas las muertes durante este período fueron potencialmente causadas por el COVID-19. La tasa de mortalidad varia por etnia y por región (tabla suplementaria 1), siendo la tasa en Ucayali mayor al doble que la tasa en Loreto (0,118% vs. 0,047%) y sustancialmente mayor en caseríos mestizos que en comunidades indígenas (0,118% vs. 0,061%).

Al comparar la proporción de comunidades con casos y muertes por COVID-19 entre comunidades indígenas y caseríos mestizos no se observa ninguna diferencia sustancial en la presencia de casos y mortalidad potencialmente debidos al COVID-19 en julio; salvo una excepción, una proporción más grande de comunidades indígenas reportó algún caso confirmado hasta julio (figura 1A). A partir de entonces, la prevalencia (algún caso en julio, sea presunto o confirmado; algún caso en agosto) e incidencia (caso nuevo en agosto) se incrementó en comunidades indígenas, lo que sugiere que en agosto las comunidades indígenas se vieron más seriamente afectadas que los case­ríos mestizos. De manera similar, al comparar la proporción de comunidades con casos y muertes entre las comunidades de Loreto y Ucayali se puede observar que la presencia de algún caso o alguna muerte debidos al COVID-19 fue más común en Ucayali que en Loreto (figura 1B).

**Figura 1:** Prevalencia y propagación de COVID-19 en comunidades rurales amazónicas por etnia y por región durante los primeros meses de la pandemia en Perú.

Nota: Algún caso incluye todo caso, incluidos los casos confirmados y casos sospechosos. Hasta julio se refiere algún caso o muerte entre mediados de marzo y julio 2020. En julio (o en agosto) se refiere a la presencia de casos al momento de nuestra encuesta de base en julio 2020 (o encuesta de seguimiento en agosto). Para algunas variables, el número de observaciones es menor que el número de comunidades en la muestra (reportadas en la figura 1) debido a valores faltantes.

### Medidas de protección

Al momento de la encuesta de base, de entre las medidas de protección, solamente el lavado de manos había sido adoptado de manera generalizada (sí) o parcialmente (más o menos) en la mayoría de las comunidades (96,0%); el uso de mascarilla y las medidas de distanciamiento social, evitar saludos físicos, reuniones sociales y viajes, mantener una distancia mínima, quedarse en casa y restringir el ingreso a la comunidad, no estaban siendo adoptados en entre el 10,0% y 27,0% de las comunidades (figura 2A). El lavado de manos, uso de mascarillas y el establecimiento de restricciones de ingreso a las comunidades fueron adoptados de manera más generalizada que otras medidas de protección (~60,0% vs. ~40,0% de las comunidades).

**Figura 2:** Medidas de protección en comunidades rurales amazónicas durante los primeros meses de la pandemia en Perú.

Notas: En los paneles A, B, C y D *Mantener distancia* corresponde mantener una distancia suficiente de al menos 1 metro; *Quedarse en casa* corresponde a no salir de su casa a menos que sea necesario*; Evitar reuniones* corresponde a evitar reuniones con muchas personas; *Evitar viajar* corresponde a evitar que la gente viaje a otros lugares (comunidades rurales o la ciudad); *Restringir el acceso de fuera* corresponde a limitar el acceso a la comunidad de gente de fuera. Las otras medidas de protección son obvias.

En los paneles A y B, *si*, *no* y *más o menos* se refieren al grado de adopción de cada medida de protección en base a las preguntas originales de las encuestas: *si* corresponde a adopción generalizada, *más o menos* a adopción parcial y *no* a la no adopción de la medida. En los paneles C, *Sin incidencia y Con incidencia* se refieren a la presencia de casos de COVID-19 en la comunidad al momento de la encuesta de base (julio de 2020); *Sin nueva incidencia* y *Con nueva incidencia* se refieren a la presencia de casos nuevos de COVID-19 en la comunidad (durante los 7 días previos a la encuesta de seguimiento). El número de observaciones es menor que el número de comunidades en la muestra (reportadas en la figura 1) debido a valores faltantes.

Para el momento de la encuesta de seguimiento, el lavado de manos y el uso de mascarilla se hicieron más comunes en relación con la encuesta de base en julio, pero al mismo tiempo, todas las medidas de distanciamiento social disminuyeron en su adopción (figura 2B).

Los datos de la encuesta de base indican que las medidas de protección entre mediados de marzo y julio fueron más comunes en comunidades sin algún caso de COVID-19 (incluidos casos presuntos) en julio (figura 2C). En agosto, sin embargo, las medidas de distanciamiento social fueron más comunes en comunidades que reportaron algún nuevo caso (figura 2D), posiblemente para evitar el contagio.

### Asistencia

Para julio de 2020, la gente en el 97,0% de las comunidades había recibido alguna asistencia desde mediados de marzo. Mientras que la asistencia alimentaria (víveres) y monetaria (bonos) fue común (más de 80% de las comunidades), fueron pocas las comunidades que para entonces habían recibido mascarillas, desinfectante, jabón o medicinas. El estado fue la fuente de asistencia más común (96,0%) siendo muy escaso el apoyo de otras fuentes (federaciones indígenas, organizaciones no gubernamentales y organizaciones internacionales).

Cuando se examina de manera conjunta los datos sobre la asistencia recibida con aquellos sobre el COVID-19 se puede observar que la asistencia monetaria fue más frecuente en comunidades con mortalidad potencialmente debida al COVID-19 entre mediados de marzo y julio, y en comunidades con algún caso de COVID-19 (incluidos casos presuntos) en julio; no hubo tal diferencia en la recepción de asistencia no monetaria (principalmente víveres) (figura suplementaria 3).

## DISCUSIÓN

Los resultados de este estudio observacional longitudinal sobre COVID-19 indican que, a pesar de las medidas de cuarentena y las dificultades de acceso en la región, el COVID-19 se propagó ampliamente por las comunidades rurales en Loreto y Ucayali y la mortalidad varió por región y por etnia. La adopción de medidas de protección y la asistencia social afectaron el contagio.

Mas del 90% de las comunidades reportaron algún caso de COVID-19. Inicialmente el COVID-19 se presentó más en los caseríos mestizos, pero después tuvo mayores efectos en las comunidades indígenas. Las comunidades indígenas tienden a encontrarse en zonas más remotas lo cual indica que las dificultades en el acceso pueden haber servido como una barrera que demoró mas no eliminó el contagio hacia esas zonas ^21^. Se encontró también que un porcentaje importante (45%) de las muertes registradas durante la etapa temprana de la pandemia fueron causadas potencialmente por el virus. No obstante, la tasa de mortalidad fue menor que en la ciudad (de 0,270%, basada en los datos de MINSA para Loreto y Ucayali; tabla suplementaria 1) y la media nacional (0,186%), reportándose muertes en solo 15,0% de las comunidades, más comúnmente en caseríos mestizos. La baja tasa de mortalidad reportada aquí es consistente con otros de los estudios sobre pueblos indígenas en Ucayali y Amazonas basados en datos oficiales de MINSA o DIRESA ^13^^,^^22^, los cuales dan un respiro ante la preocupación inicial que se tuvo, sobre todo tomando en cuenta las vulnerabilidades preexistentes de estas poblaciones y las enormes dificultades para proveer servicios de salud adecuados en zonas rurales aún en tiempos normales ^4^^,^^23^^-^^25^. Sin embargo, no se debe descartar la importancia cultural de la muerte de varios ancianos indígenas ^26^. Estudios sobre los factores que ayudaron a prevenir el contagio y la mortalidad en comunidades rurales serán de gran utilidad en el futuro. En particular, se necesita entender mejor el potencial de la medicina tradicional y del conocimiento ecológico local para complementar las políticas públicas sobre pandemias y para mejorar la salud humana en las comunidades rurales ^27^^-^^29^.

Este estudio apunta también a diferencias estructurales entre las comunidades rurales que ameritan atención seria. En comparación con Loreto, las comunidades en Ucayali tuvieron una tasa de mortalidad por COVID-19 muy superior (0,118% vs. 0,047%) y una mayor prevalencia tanto en casos como en mortalidad. Inicialmente, la tasa de mortalidad debida al COVID-19 fue superior en caseríos mestizos que, en las comunidades indígenas, aunque el COVID-19 se volvió más frecuente en las comunidades indígenas durante el segundo brote. Es necesario realizar más estudios para comprender mejor los factores que subyacen tales diferencias, aunque es claro que todas las comunidades rurales (tanto indígenas como mestizas) requieren atención más seria y mayor participación para mejorar su salud ^14^^,^^27^^-^^29^.

En términos de medidas de protección, se encontró también una mayor tasa de adopción del lavado de manos y uso de mascarilla en relación con otras medidas de distanciamiento social (60,0% vs. 40,0%), lo cual indica que el distanciamiento social es difícil en comunidades rurales, posiblemente debido a normas sociales y culturales; algo que también ha sido reportado en otros estudios ^12^^,^^21^. Es necesario conocer mejor el potencial y las barreras para la adopción de medidas de protección socialmente costosas para reducir el contagio, como en este caso, el distanciamiento social y el evitar reuniones. Se necesitan más estudios para identificar los factores que afectan la adopción de medidas de protección, que pueden servir de guía para intervenciones que promuevan su adopción y que respeten las normas socioculturales subyacentes ^12^^,^^27^^,^^30^.

Si bien los programas de asistencia estatal llegaron a la mayoría de las comunidades y ayudaron a la población, la recepción de bonos estatales (82,0% recibieron bonos) estuvo asociada con muertes potenciales por COVID-19 (11,0% más de las comunidades), sin observarse la misma relación con la provisión de ayuda no monetaria (principalmente víveres). Esto se atribuye, no al tipo de asistencia en sí, sino al modo de prestación de la misma. Mientras que los víveres fueron entregados directamente en las comunidades, la gente tuvo que viajar a Iquitos, Pucallpa, o capitales distritales para cobrar su bono. Como otros estudios Loreto y Ucayali han sugerido, estos viajes contribuyeron de manera imprevista al contagio en comunidades rurales ^4^^,^^12^^,^^13^^,^^21^. Se requiere de políticas públicas alternativas, tanto nuevas políticas como mejoras de las políticas actuales, a saber: protocolos alternativos para proporcionar asistencia social, tales como dinero móvil y las plataformas itinerantes multifuncionales del Ministerio de Inclusión Social, y el suministro de recursos de tecnología de información y comunicación en zonas rurales. Se necesita más investigación aplicada para identificar enfoques prometedores y factibles en el contexto local.

Se identifican cuatro principales limitaciones del presente estudio. Primero, la muestra de comunidades no es representativa del área de estudio debido a las dificultades para contactar a todas las comunidades meta por teléfono móvil; problemas de validez externa han sido comunes con encuestas telefónicas durante la pandemia. Segundo, no se puede descartar la posibilidad de errores de medición, aunque las encuestas telefónicas a nivel comunitario reducen la posibilidad de sesgos en los reportes (p. ej. sesgo de conveniencia social) en relación con encuestas telefónicas de hogares. Tercero, debido al escaso acceso a establecimientos de salud y a pruebas de COVID-19, el número de casos confirmados de COVID-19 reportados en las encuestas es incompleto. El número de muertes reportadas debería de ser más fiable, aunque las percepciones de los participantes sobre si las muertes fueron causadas por COVID-19 pueden ser imprecisas; por ende, el estudio se enfoca en la presencia de casos y muertes en las comunidades como medidas crudas de la situación. Los datos capturados no permiten estudiar las dinámicas a niveles de hogares o de individuos. Finalmente, evolución de COVID-19 no pudo ser capturada en algunas comunidades por la pérdida de comunidades en la encuesta de seguimiento.

En conclusión, el COVID-19 se propagó ampliamente por las comunidades rurales en la selva, aunque la tasa de mortalidad fue menor que en la ciudad, siendo sus impactos mayores en las comunidades indígenas y en las de Ucayali. Barreras socioculturales a la adopción de medidas de distanciamiento social y la necesidad de viajar para cobrar los bonos del estado contribuyeron al contagio en estas poblaciones. Aunado a la necesidad de un enfoque de salud intercultural y más inclusivo para fortalecer el sistema de salud rural ^22^^,^^24^^,^^29^^,^^30^, se requiere conocer mejor los factores que afectan la adopción de medidas de protección, así como repensar los modos de distribución de programas de ayuda del estado que permitan evitar el contagio en pandemias futuras. En términos más generales, deberá priorizarse establecer sistemas confiables para la captura de datos que reflejen las condiciones en comunidades rurales para poder informar políticas efectivas.
